# Ophthalmic complications during the dengue epidemic in Reunion Island in 2020: a case series and review of the literature

**DOI:** 10.1186/s12879-023-08432-4

**Published:** 2023-08-02

**Authors:** Digé Mbu-Nyamsi, Muriel Vincent, Mariane Perez-Fontana, Anne-Laurence Best, Charles Mesnard, Fréderic Villeroy, Aurélie Foucher, Loic Raffray, Cécile Saint-Pastou Terrier, Antoine Bertolotti

**Affiliations:** 1 Département de maladies infectieuses-médecine interne-dermatologie, CHU de La Réunion, Saint Pierre, La Réunion France; 2 Santé Publique France, Océan Indien, Saint Denis, La Réunion France; 3 Département d’ophtalmologie, CHOR, Saint Paul, La Réunion France; 4 Département d’ophtalmologie, CHU de La Réunion, Saint Pierre, La Réunion France; 5 Département d’ophtalmologie, CHU de La Réunion, Saint Denis, La Réunion France; 6 Département de médecine interne, CHU de La Réunion, Saint Denis, La Réunion France; 7 Unité Mixte de Recherche Processus Infectieux en Milieu Insulaire Tropical (PIMIT), Université de La Réunion, INSERM UMR 1187, CNRS 9192, IRD 249, Plateforme CYROI, Sainte Clotilde, La Réunion France; 8 CHU de La Réunion, CIC-INSERM1410, Saint Pierre, La Réunion France

**Keywords:** Dengue, Reunion island, Maculopathy, Scotoma, Arbovirosis, Ophthalmology, Dengue, Reunion Island

## Abstract

**Introduction:**

Dengue is an arboviral disease transmitted by the dengue virus, whose vectors are *Aedes aegypti* and *Aedes albopictus*. The acute phase with its cohort of well-known symptoms is usually spontaneously favorable. Since 2020 in Reunion Island, a new symptom has appeared: the ocular damage of dengue fever, which has already been described in South Asia and South-East Asia. We therefore decided to describe the clinical, biological, ophthalmological, therapeutic, and outcomes of patients with ocular manifestations during dengue fever in Reunion Island in 2020.

**Patients and methods:**

This was a retrospective observational study. Patients were included from January 2020 to August 2020 and then reassessed by teleconsultation 1 year later. The patients were identified from the French public health surveillance network by all ophthalmologists on the island. Medical data were collected directly from medical records.

**Results:**

Twenty-eight patients were included. The mean age was 41.9 years. Ocular involvement occurred approximately 9.2 days after the onset of dengue symptoms. The main symptoms were scotoma (71.4%) and sudden decrease of visual acuity (39.2%). Eighteen patients (64.2%) had macular involvement. Fourteen patients were treated with oral or intravenous corticosteroids. Twenty-two (78.5%) patients were evaluated by telephone one year later. Scotoma and decreased visual acuity persisted in 15 patients. Thirteen patients (59%) were bothered by night driving, 32% of patients had reading difficulties and 27% of patients became sensitive to prolonged exposure to screens.

**Conclusion:**

Ocular complications of dengue require early and collegial management to limit the risk of long-term sequelae. Further studies on the characteristics and complications of dengue fever are needed to better understand this disease.

**Supplementary Information:**

The online version contains supplementary material available at 10.1186/s12879-023-08432-4.

## Introduction

Dengue is a mosquito-borne disease transmitted by *Aedes aegypti* and *Aedes albopictus*. Dengue virus (DENV) belongs to *Flaviviridae* and is divided into four antigenically-related serotypes of virus [1, 2]. Dengue infection is characterized by an acute onset of fever associated with symptoms of asthenia, arthralgia, myalgia, nausea, vomiting, abdominal pain, and skin rash. The evolution of dengue is generally favorable but severe forms can occur. They are represented by (i) hemorrhagic manifestations associated with thrombocytopenia, usually occurring after 5 to 8 days of fever onset, (ii) severe plasma leakage and (iii) organ failure [3].

Apart from these well-known multi-systemic manifestations, dengue can also affect eyes. Reunion Island, a French overseas territory where chikungunya was rife in 2005–2006 [4], has since 2017 been the scene of an increasingly important resurgence of dengue fever [5]. During the year 2020, a total of 16,100 cases of dengue fever were reported, mostly during the austral summer, with a peak in April. Since 2020, various ophthalmologists on the island have warned of the emergence of ocular damage following dengue infection. The wide clinical panel of ophthalmic complications of dengue fever includes anterior uveitis, retinal vasculitis and oedema, exudative retinal detachment, optic neuritis, ischaemic optic neuropathy, branch retinal artery occlusion, and retinal pigment epithelial disturbance with a preponderance for maculopathy [6]. These complications has been described principally in South Asia and Southeast Asia through short retrospective studies [7–9]. Prevalence of ocular manifestations reaches up to 10%, and as many as 5–8% (depending on the study) of patients may have a worse long term visual prognosis [10, 11].

We describe herein the clinico-biological signs and 1 year evolution of the patients with ocular complication of dengue acquired during the 2020 outbreak in Reunion Island.

## Patients and methods

### Study design, population and statistical analysis

We conducted a multicentric retrospective descriptive study. Data was collected from the regional surveillance network of the Reunion public health agency and medical files. Information on clinical and functional impact of ocular complication of dengue was collected during a teleconsultation one year after their initial management, as part of the clinical follow-up of these patients. The diagnosis was confirmed either by positive reverse transcription Polymerase chain reaction or IgM positive serology followed by IgG positive 3 weeks later (Panbio serologic test from Abbott). Descriptive statistics were computed for continuous data (mean ± standard deviation (SD) and for categorical data (frequency and proportion).

### Ethics approval and consent to participate

In accordance with French regulations, this study did not require an Ethics Committee (article R1121-1, decree n°2017–884 of 9 May 2018—art.2), but the study was conducted according to the Declaration of Helsinki and the reference methodology MR-004 of the National Commission for Information Technology and Liberties (CNIL) and was approved by the Regional Health Agency. Indeed, by sharing data related to diseases (in this case dengue) with the regional team of Santé Publique France, this study has supported the government agency in its mission to survey public health and improve decision-making. Informed consent was obtained from all participants and data were treated anonymously.

## Results

Twenty-eight patients were included during the epidemic of austral summer, from January 2020 to August 2020. All cases were confirmed by PCR or serology. The serotype could only be identified in 50% of the cases, and all were type 1 strains (Table 1).

**Table 1:** Characteristics of patients and ocular manifestations, Reunion Island. 2020 (*N* = 28)

Variable (*N* = 28)	Number (%)
Socio-demographical	
Mean age (years) [SD]	41.9 [15.9]
Men	8 (28.5)
Women	20 (71,4)
Clinical symptoms	
Mean delay (days) [SD]	9.2 [5.0]
Decrease of visual acuity	11 (39.2)
Scotoma	20 (71.4)
Macula damage	18 (64.2)
Macula oedema	7 (25.0)
Foveolitis	7 (25.0)
Papilla damage	8 (28.5)
OCT: retinal thickening	11 (39.3)
OCT: hyper reflectivity	9 (32.1)
Fundus: hemorrhage	3 (10.7)
Fundus: cotton-wool spots	3 (10.7)
Uveitis	6 (21.4)
Biological:	
Serotype identified: type 1	14 (50.0)
Secondary dengue	3 (10.7)
Positive RT-PCR	23 (82.14)
IgM Positive serology^a^	5 (17.5)
Thrombocytopenia	4 (14.2)
Treatment	
Oral steroids	9 (32.1)
Intravenous steroids	14 (50.0)

*C4* Complement component 4, *PCR* Polymerase chain reaction, *OCT* Optical Coherence Tomography, *SD* Standard deviation

^a^Followed by positive IgG 3 weeks later

### Ophthalmic damage

**Fig. 1:**
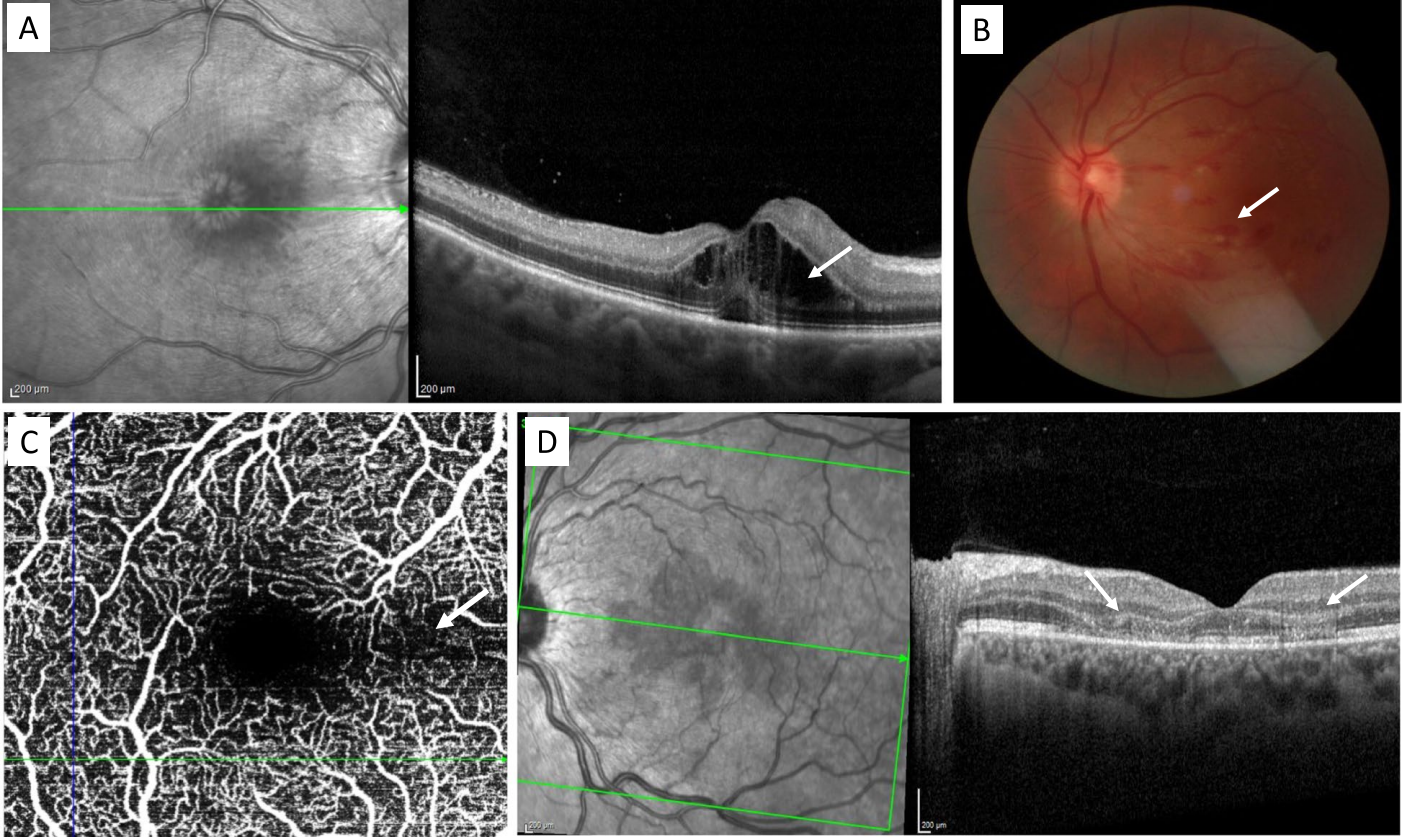
Ocular anomalies during dengue – **A** OCT of the macula: cystoid macular oedema (white arrow) **-B** Retinography of a left eye showing flare hemorrhages on the posterior pole. **C** OCT angiography: perfusion defect in the deep capillaries of the macula in the Para foveolar region (white arrow) **-D** OCT of the macula (white arrow): hyper reflectivity of the outer plexiform layer and nuclear layer of retinal cells and fragmentation of ellipsoid zone. OCT: Optical Coherence Tomography

Eye involvement appeared on average 9.2 days (SD: 5.0, min: 5.0 – max: 21,0) after the onset of dengue symptoms. The main symptoms were scotoma (71.4%) and sudden decrease in visual acuity (39.2%). Patients with maculopathy following dengue fever presented an altered OCT of the macula with a cystoid macular oedema (Fig. 1).

Regarding the treatment, 46.4% of the patients were treated with corticosteroids (Table 1).

### Evaluation at one year

An internist doctor evaluated the vision impairment after one year with a binary response questionnaire, via teleconsultation. Twenty-two patients (78.5%) answered but the other 6 patients were lost to follow-up. Fifteen patients (68%) complained of persistent scotoma. Thirteen patients were bothered by night driving, 7 had reading difficulties and 6 became sensitive to prolonged exposure to screens (Table 2).

**Table 2:** Ocular manifestations at one year, Reunion Island. 2020 (*N* = 22)

Variable (*N* = 22)	Number (%)
Symptoms	
Persistent scotoma	15 (68.2)
Sequelae of decreased visual acuity	15 (68.2)
Ocular surgery	1 (4.5)
Ocular pain	3 (13.6)
Ocular tiredness	13 (59.0)
Ocular redness	2 (9.0)
Ocular dryness	4 (18.0)
Ocular itching	4 (18.0)
Feeling of ocular foreign object	2 (9.0)
Lacrimation	2 (0.0)
Impact	
Wearing of glasses	9 (41.0)
Working	5 (23.0)
Night driving	13 (59.0)
Reading	7 (32.0)
Screen exposure	6 (27.0)
Loss of vision	1 (4.5)

## Discussion

Since 2017, Reunion Island has witnessed dengue outbreaks that have become more and more important each year, until 2022. This study describes one of the largest series of dengue cases with ophthalmologic complications.

The mean age of the patients in this study was higher (41.9 years) than in a systematic review, (33.0 years) [9] and in our review of cases report with available data (32.7 years, min 6 years – max 70, Additional file 1: Appendices 1–2). It could be related to the recent appearance on Reunion Island of these epidemics in contrast to Asian countries which are endemic areas for this arbovirus. Ophthalmologic symptoms occurred 9.2 days after the onset of dengue symptoms; a delay higher than in the literature (7.0 days) [12]. However, symptoms have appeared months later in other studies [2, 9, 13]. As found in certain studies, sudden decrease of the visual acuity and scotoma are the two main ocular symptoms [7, 14–17]. Eighteen patients had macula involvement including several abnormalities: retinal thickening on OCT (39%), hyperreflectivity (32%), macular edema (25%) and foveolitis (25%). One study identified 3 groups to describe macular involvement: diffuse retinal thickening, cystoid macular edema and foveolitis [18].

Some hypotheses could be raised to explain this specific injury: (i) Firstly, the NS1 antigen of DENV has a major implication in the acute phase of dengue fever. In the eye, it makes a substantial contribution to inflammation by activating macrophages and peripheral blood mononuclear cells via the Toll-like 4 receptor [19]. In addition, it activates the complement system (specifically C5b-C9) to lyse target cells, thereby promoting the production of pro-inflammatory cytokines [20–22]. These then contribute to endothelial hyperpermeability and vascular leakage [21]. Finally, by binding to NS1 proteins, anti-NS1 antibodies activate cellular signal transduction pathways and tyrosine phosphorylation of cellular proteins. This leads to the release of interleukin-6 and interleukin-8, as well as monocyte chemoattractant protein (MCP-1). By increasing ICAM-1, MCP-1 increases the adhesion of immune system cells to endothelial cells [23–25]. Anti-NS1 antibodies therefore also appear to contribute to ocular inflammation and cell apoptosis via nitric oxide [26].

(ii) Secondly, plasma components are important mediators during dengue infection [27] and specifically exosome fraction. Exosomes are nanoscale membrane vesicles released by almost all types of cells infected with a virus, bacteria or parasite. They serve as vectors for various mediators and participate in the organism's response to infection. Exosomes communicate with host cells through contact between exosomes and their recipient cells, via different kinds of mechanisms. Increased production of exosomes is a characteristic feature of DENV infection [28, 29]. These cargo molecules, which carry biological information (mRNA, miRNA, transcription factors) from their cells of origin, are involved in the alteration of recipient cell behavior, regulating cellular responses, stress signals and enabling infection by DENV [30]. In DENV infection with ophthalmic complications, the relevance of exosomes could be instrumental since the majority of the immune responses in severe dengue involve heavy secretion and circulation of pro-inflammatory cytokines and chemokines [28].

(iii) Thirdly, endothelial dysfunction could also be caused by antibodies produced during primary infection, known as antibody-dependent enhancement (ADE) [26]. Internalisation of DENV via the Fcγ receptor is thought to inhibit the expression of antiviral genes by suppressing type 1 interferon (IFN)-mediated antiviral responses. At the same time, however, there is a decline in regulatory T cells (Treg). The latter, limit the production of antigen-specific antibodies, thereby attenuating viral replication and the formation of ADE-mediated immunological complexes [31, 32]. The action of Tregs then leads to an increase in IL-10, which means that there is a shift from a Th1 immune response to an immune response in favour of Th-2 cells with less anti-viral activity [33, 34]. These various responses then contribute to retinal vascular occlusions, posterior uveitis and retinal haemorrhages during the critical phase of the ocular complications of FTD [31, 35]. In La Réunion, we suspect that this is one of the main causes, as these cases only appeared during the 3rd consecutive epidemic, when DENV-1 was in the majority. Indeed, in 2018, at the beginning of the outbreak, DENV-2 was the first serotype detected on the island, then DENV-1 started being detected in 2019 (12%). During the course of the epidemic in 2020, DENV-1 rapidly became the dominant serotype until 2022 (84% in 2020 then 100% in 2021) [36].

(iv) Fourthly the dengue serotype and certainly the dengue genotype also seem important to consider in this phenomenon, as the majority of publications of ophthalmological damage come from cases affected by dengue type 1 and originating in Asia [14, 37]. We were not able to genotype DENV-1 strain in our patients, but DENV-1 was genotyped in 2019, 2020 and 2021 in other patients in Reunion Island. These sequences showed greatest similarity (99.5% identity) to one isolate from Sri Lanka in 2020 (Accession number OL752439.1) and two sequences from China in 2016 (Accession numbers MN933661 and MN933663) [38].

(iv) Lastly, leukopenia and hypoalbuminemia could predispose to opportunistic infection of the ocular tissues and increased vascular permeability, respectively [39].

Treatment with corticosteroids had been initiated in 14 patients in collaboration with the ophthalmologist. Its duration and dosage were related to the severity of the disease. The use of this treatment or intravenous immunoglobulins was mainly reserved for severe cases [17, 18]. None of the 28 patients received immunoglobulins.

The evolution of ocular involvement is usually considered to be favorable 2–4 weeks later [9, 17, 40, 41]. However, 1 year later, visual acuity remains diminished in some patients and the scotoma persists. Dengue-related macular damage could therefore become a public health issue.

The main limitations of our study are, firstly, the retrospective nature of the study, implying the presence of missing data and preventing the identification of relevant results allowing their extrapolation, and secondly, the absence of collegiality between the different collaborators which may have prevented a homogeneous management of patients. Other studies are now underway to better explore these phenomena in Reunion Island.

## Conclusion

Ocular involvement in dengue remains poorly described because of its rarity. This study alerts health professionals to the impact of this disease over a one-year period on an island where this arbovirus is currently becoming endemic. Prospective studies are therefore necessary to better understand the pathophysiological mechanisms of dengue, to standardize therapeutic management and to better prevent complications.

## Supplementary Information

**Additional file 1: Appendix 1.** Ophthalmic complication in dengue, review of literature.** Appendix 2.** Bibliography of the literature review.

## Data Availability

The data underlying this article will be shared on reasonable request to the corresponding author.
