# Supplementary material for: Ophthalmic complications during the dengue epidemic in Reunion Island in 2020: a case series and review of the literature
Source: BMC Infect Dis. 2023 Aug 2;23:506. doi: 10.1186/s12879-023-08432-4 (PMC10394947; doi:10.1186/s12879-023-08432-4)
Supplement: Supplementary file 1 — Additional file 1: Appendix 1. Ophthalmic complication in dengue, review of literature. Appendix 2. Bibliography of the literature review. [file 12879_2023_8432_MOESM1_ESM.docx]

**SUPPORTING INFORMATION**

**Ophthalmic complications during the dengue epidemic in Reunion Island in 2020: a case series and review of the literature**

Digé Mbu-Nyamsi^1^ (MD), Muriel Vincent^2^ (MD), Mariane Perez-Fontana^3^ (MD), Anne-Laurence Best^4^ (MD), Charles Mesnard^3^ (MD), Fréderic Villeroy^5^ (MD), Aurélie Foucher^1^ (MD), Loic Raffray^6,7^ (MD, PhD), Cécile Saint-Pastou Terrier^1^ (MD,MSc), Antoine Bertolotti^1,8^ (MD, PhD)

^1^CHU de La Réunion, département de maladies infectieuses-médecine interne-dermatologie, Saint Pierre, La Réunion

^2^Santé Publique France, Océan Indien, La Réunion

^3^CHOR, département d’ophtalmologie, Saint Paul, La Réunion

^4^CHU de La Réunion, département d’ophtalmologie, Saint Pierre, La Réunion

^5^CHU de La Réunion, département d’ophtalmologie, Saint Denis, La Réunion

^6^CHU de La Réunion, département de médecine interne, Saint Denis, La Réunion

^7^Unité Mixte de Recherche Processus Infectieux en Milieu Insulaire Tropical (PIMIT), Université de La Réunion, INSERM UMR 1187, CNRS 9192, IRD 249, Plateforme CYROI, Sainte Clotilde, La Réunion, France

^8^CHU de La Réunion, CIC-INSERM1410, La Réunion

Appendix 1 - Ophthalmic complication in dengue, review of literature (inception to October 2022 on pubmed)

| **Author, year** | **Study type** | **Gender W / M** | **Mean age, (years)** | **Number of patients** | **Eyes Number** | **Ocular symptoms** | **Ocular signs** | **Treatment** | **Follow-up** | **Outcome** |
| --- | --- | --- | --- | --- | --- | --- | --- | --- | --- | --- |
| **Khan et al, 2020** ^1^ | Case report | 1 M | 19 | 1 | 2 | Bilateral visual loss |  | Corticosteroids | No | Improvement |
| **Haritoglu et al, 2002** ^2^ | Case report | 1 W | 21 | 1 | 2 | Bilateral decreased visual acuity | Bilateral exudative maculopathy and small hemorrhages in the nerve fiber layer | No treatment | Weeks | Improvement |
| **Li et al, 2015** ^3^ | Prospective case series | 3 W / 2M | 38 | 5 | 9 | Central/paracentral scotoma | Acute macular  neuroretinopathy, intraretinal hemorrhages, central artery occlusion | No treatment | Every two weeks for 6 months |  |
| **Nagaraj et al, 2014** ^4^ | Case report | 1 M | 20 | 1 | 1 | Pain and swelling |  | Antibiotics |  | No perception of light in the left eye |
| **Filho et al, 2008** ^5^ | Case report | 1 W | 67 |  |  | Loss of vision, intense ocular pain, eye watering, photophobia, and redness of both eyes | Elevated intraocular pressure, bilateral acute angle closure glaucoma | Intravenous mannitol.  Timolol, pilocarpine and prednisolone  Eye drops |  | Improvement |
| **Boo et al, 2017** ^6^ | Case report | 1 W | 38 | 1 | 1 | Blurring of vision, reduced visual acuity | Bilateral optic neuritis with right eye macular oedema and foveolitis | Cortisteroids | 1 month | Improvement |
| **De Amorim Garcia et al, 2006** ^7^ | Case report | 1 M | 42 | 1 | 2 | Bilateral low visual acuity | Bilateral stellar neuroretinitis | Analgesics | 2 months | Improvement |
| **Nainiwal et al, 2005** ^8^ | Case report | 1 W | 14 | 1 | 1 | Diminution of vision in  Right eye | Bilateral vitreous hemorrhage | Vitrectomy in the right eye | 8 weeks |  |
| **Kanungo et al, 2008** ^9^ | Case report | 1 W | 28 | 1 | 1 | Blurring of the inferior visual field in the right eye | Branch retinal artery occlusion | No treatment | 3 months | Improvement |
| **Puthalath et al, 2021** ^10^ | Case report | 1 W | 30 | 1 | 1 | Diminution of vision in the left eye | Retinal hemorrhages, foveolitis | Oral steroids |  | Recovery |
| **Velaitham et al, 2016** ^11^ | Case report | 1 W | 41 | 1 | 1 | Blurring of vision, decrease of visual acuity | Central retinal vein occlusion, proliferative retinopathy | Panretinal photocoagulation |  | No recovery |
| **Veloso et al, 2015** ^12^ | Case report | 1 W | 54 | 1 | 1 | Blurred vision and metamorphopsia in 1 eye | Subretinal fluid and focal hemorrhage in the macula,  Choroidal neovascularization | Intravitreal injections of ranibizumab | Regular follow up | Recovery |
| **Kamal et al, 2018** ^13^ | Case report | 1 M | 33 | 1 | 1 | Pain, redness, and proptosis in 1 eye | Conjunctival chemosis and congestion, corneal edema, exposure keratopathy inferiorly, panophthalmitis | Topical and intravenous antibiotics, evisceration of the right eye |  |  |
| **Yadav et al, 2017** ^14^ | Case report | 1 W | 35 | 1 | 2 | Decrease of visual activity, scotoma | Yellow deep choroidal lesions at macula, multiple small, hard exudates along papillomacular bundle, and hyperemic disc, bilateral choroiditis | Oral prednisolone 60 mg/day | 2 weeks | Recovery |
| **Gupta et al, 2011** ^15^ | Retrospective study | 1 W / 2 M | 29 | 3 | 5 | Diminution of vision | Retinal hemorrhages in macular, retinal edema, | One patient had 1 mg/kg oral steroids, no treatments for the others | 6 weeks | Recovery |
| **Ooi et al, 2016** ^16^ | Case report | 1 M | 37 | 1 | 2 | Bilateral clouding of vision, central and paracentral scotomas | Intraretinal hemorrhage, panuveitis | Topical steroids and cycloplegia | 1 month | Recovery |
| **Kamoi et al, 2018** ^17^ | Case report | 1 W | 60 | 1 | 1 | Subconjunctival hemorrhage, pain | Necrotizing scleritis | Systemic and topical steroids, antibiotics | 18 years | Recovery |
| **Juanarita et al, 2012** ^18^ | Case report | 1 W | 24 | 1 | 2 | Bilateral loss of vision | Retinal hemorrhages, elevation of fovea area, subretinal fluid | No treatment, observation | 2 months | Recovery |
| **Tabbara et al, 2012** ^19^ | Case report | 1 M | 32, 43 | 2 | 4 | Acute loss of vision | Retinochoroiditis, vasculitis, cotton-wool spots, and retinal  hemorrhages | Oral nonsteroidal anti-inflammatory | 6 weeks and 2 months | Recovery |
| **Tan et al, 2007** ^20^ | Case report | 1 W | 37 | 1 | 2 | Bilateral  Blurring of vision with metamorphopsia | Bilateral retinal vasculitis, bilateral macular oedema, central  Foveal thickening | Anti-inflammatory agents | 10 weeks | Recovery |
| **Bascal et al, 2007** ^21^ | Retrospective study | 28 W / 35 M | 28.8±11.4 | 41 | 71 | Blurring, scotoma | Intraretinal hemorrhages, | Intravenous, oral, topical steroids, intravenous immunoglobulins | 5.4±4.8 months | All had good visual  Recovery |
| **Loh et al, 2008** ^22^ | Retrospective review | 2 W / 4 M | 19.8 | 6 | 10 | Acute visual loss | Yellow-orange lesion at the fovea, foveolitis | Intravenous or oral steroid, intravenous immunoglobulins. | 9.7 months | All had good visual  Recovery |
| **Akanda et al, 2018** ^23^ | Case report | W | 43 | 1 | 2 | Scotoma |  | Oral steroid | 1 months | Persistent scotomas |
| **Preechawat et al, 2012** ^24^ | Case report | M | 15 | 1 | 1 |  | Optic disc edema, peripapillary and prepapillary  hemorrhages, and cotton wool spots, intraretinal hemorrhages, non-ischemic Central Retinal Vein Occlusion (delayed venous filling, areas  of capillary drop out, dilated, tortuous  retinal veins) | Intraveinous and oral steroid, aspirin | 10 months | Recovery |
| **Kapoor et al, 2006** ^25^ | Study | 63% males | 31.3 | 134 |  | No complaints | Subconjunctival hemorrhages, superficial retinal hemorrhages, cotton wool spots, | No treatments | 2-8 weeks | All had good visual recoveryecovery |
| **Lim et al, 2004** ^26^ | Retrospective case series and literature review | 5 W / 1 M | 35,8 | 6 |  | Blurring of vision, scotoma |  | Topical, periocular, oral steroïd |  |  |
| **Chan et al, 2006** ^27^ | Retrospective case series | 7 W / 6 M | 31,7 | 13 | 22 | Central vision impairment (12), blurring of vision, decrease of visual acuity | Macular edema and blot hemorrhages (10), cotton wool spots (1), retinal vasculitis (4), exudative retinal detach- ment (2), and anterior uveitis (1) | No treatment for 11 patients, 2 patients received systemic steroid | 12 weeks | Recovery with residual scotoma |
| **Sanjay et al, 2008** ^28^ | Case series | 2 W / 1 M |  | 3 | 6 | Unilateral blurring of vision, impaired color vision, optic disc  Swelling, retinal edema, cotton  Wool spots, retinal hemorrhage | Optic neuropathy retrobulbar optic neuritis  Bilateral  Maculopathy, central scotoma in  The right eye and paracentral defects in the left eye. | No treatment  IV and oral steroid | 6 weeks – one year | No recovery, blindness (1)  Persistent impaired colour vision,  And a paracentral scotoma in the left eye (2) |
| **Tavassoli et al, 2016** ^29^ | Case report | 1 W | 26 | 1 | 1 | Reduction in vision and central visual loss | Retinal hemorrhage superior to the macula, swollen pale retina, retinal ischemia |  |  | Recovery persistent central scotoma |
| **Arya et al, 2019** ^30^ | Case report | 1 M |  | 1 | 1 | Pain, watering, redness, swelling and loss of vision | Subconjunctival hemorrhage and chemosis, panophthalmitis with intraocular hemorrhage were made | Eye evisceration |  | A customized ocular prosthesis was fitted |
| **Saranappa et al, 2012** ^31^ | Case report | 1 W | 6 | 1 | 1 | Pain, Vision reduced to perception of light, | Panophthalmitis | NA | NA | Visual loss |
| **Shivanthan et al, 2012** ^32^ | Case report | 1 M | 29 | 1 | 1 | Binocular  Diplopia | Symptomatic right lateral rectus palsy | NA | 3 months | Recovery |
| **Fang et al, 2017** ^33^ | Case report | 1 W | 18 | 1 | 2 | Blurred vision, central scotoma | Darkish red lesions at the posterior pole, disruption of the outer neurosensory retina involving the outer limiting membrane, the myoid and ellipsoid zone as well as the outer segments of the photoreceptors | Oral steroid | 2 months | Recovery |
| **Ramananda et al, 2018** ^34^ | Case series | 3 M |  | 3 | 5 | Patient 1: diminution of vision in both eyes, pain, protrusion of the right eye, platelet transfusion no perception of light in both eyes  Patient 2: painful loss of vision  And forward protrusion of both eyes, platelet transfusion, no perception of light in both eyes  Patient 3: painful diminution of vision, redness  And blurring, | Patient 1 :  -RE post platelet  Transfusion panophthalmitis  -LE: endophthalmitis  Patient 2:  -RE: central corneal endothelial plaque with anterior  Chamber cellular reaction and hypopyon  -LE:  Bilateral  Post platelet transfusion panophthalmitis  Patient 3: no perception in the right eye. Swelling, chemosis, congestion, and anterior  Chamber and vitreous exudation | Patient 1: empirical meningitic doses of intravenous broad-spectrum  Antibiotics and topical broad-spectrum antibiotic drops, cycloplegic  Drops, and steroid drops  Patient 2: same treatment as patient 2 |  | Patient 1: recovery but no perception of light  In both eyes  Patient 2: perception of light in both eyes  Patient 3: intensive care unit for dengue shock syndrome,  Acute kidney injury, and right-sided frontal bleed  Causing left hemiparesis |
| **Quek et al, 2009** ^35^ | Case report | 1 W | 39 | 1 | 2 | First dengue: bilateral  Blurring of vision  Second dengue; visual disturbance | First dengue: macular cottonwool  Spots, bilateral flame haemorrhages, and macula  Oedema with vascular sheathing, vascular leakage in all  Quadrants including maculae, retinal periphlebitis with  Right macular branch vein occlusion and severe macula  Oedema  Second dengue: central scotoma on the right and  Patchy defects on the left eye | First dengue | First dengue: 6 months  Second dengue: 2 years | First dengue: recovery with paracentral relative scotoma  Second dengue: recovery but persistence of scotoma |
| **Chlebicki et al, 2005** ^36^ | Case series | 1 W / 3 M | 34 | 4 | 8 | Reduced visual acuity  Visual problems in the morning upon  Waking up (2)  Metamorphopsia (1)  Mild myopia (1) | Bilateral blot hemorrhages  Within the vascular arcades | All  Patients received standard supportive care  2 patients received platelet transfusion | 2 days (3)  2 months (1) | Recovery (3)  Reduced visual acuity and  Metamorphopsia even after 2 months (1) |
| **Agarwal et al, 2020** ^37^ | Case report | 1 M | 44 | 1 | 2 | Diminution of vision in both eyes | Vitritis, perivascular exudates, intraretinal hemorrhages, macular edema, peripapillary retinal whitening and cotton wool spots in both eyes suggestive of vasculitis | IV steroid but was futile, he was counseled for treatment with immunoglobulin, which the patient refused |  |  |
| **Vijitha et al, 2021** ^38^ | Retrospective study | 5 W / 18 M | 37.62 ± 18.68 | 23 | 29 | Decreased vision, eye pain, redness, discharge, bleeding from the eye, corneal clouding, proptosis, limitation of extraocular motility and diffuse scleral melt | Endophthalmitis, panophthalmitis, orbital cellulitis with panophthalmitis, isolated corneal or scleral melt, and orbital hemorrhage with panophthalmitis | Antibiotics, evisceration, vitrectomy |  | One improvement |
| **Padmanaban et al, 2018** ^39^ | Case series |  |  | 5 |  | Loss of vision | Retro-bulbar hemorrhage with hyphema-ended with globe luxation, 2. Suprachoroidal hemorrhage with globe rupture, 3. Endogenous Endophthalmitis with ring abscess going for Panophthalmitis, 4.Premacular hemorrhage resolved with some improvement in vision after hyaloidotomy, 5.Massive suprachoroidal hemorrhage |  |  |  |
| **Jyothi et al, 2018** ^40^ | Case series | 1M/2W |  | 3 | 3 | Patient 1: acute bleeding, swelling and redness  Patient 2: bleeding from the right eye, redness, pain, and swelling  Patient 3: swelling and pain | Patient 1: periorbital edema, 360° hemorrhagic chemosis, and corneal perforation at the temporal limbus with reddish-brown discharge, panophthalmitis  Patient 2: periorbital edema, 360° hemorrhagic chemosis, and central corneal perforation with brownish discharge, panophthalmitis  Patient 3: periorbital edema and erythema with 360° hemorrhagic chemosis, orbital cellulitis | Patient 1: evisceration  Patient 2: topical and parenteral antibiotics  Patient 3: topical and parenteral broad-spectrum antibiotics, mannitol, pressure bandage, and other supportive measures  (Proteus mirabilis) | Patient 1: ?  Patient 2: under follow up  Patient 3: under follow up | Patient 1: ? |
| **Chhavi et al, 2013** ^41^ | Case report | 1W | 11 | 1 | 2 | Congestion of both eyes | Bilateral lower cornea with sub-conjunctival hemorrhage of her left eye | Conservative treatment | 2 days | Recovery |
| **Chuah et al, 2017** ^42^ | Case report | 1M | 25 | 1 | 2 | Bilateral scotoma and metamorphosia | Cotton wool spots at bilateral macula and flame-shaped hemorrhage on the left, with bilateral dull foveal light reflex, dengue maculopathy | Conservative treatment | 6 months | Recovery with residual cotton wool spots |
| **Guardiola G.A et al, 2022 ^43^** | Case report | 1W | 18 | 1 | 2 | Painless paracentral scotomas | Hypopigmented macular lesions, subtle hypoautofluorescence parafoveal lesion, bilateral hyperreflectivity at he outer nuclear and photoreceptor layers | Stop oral contraceptive therapy (acute macular neuroretinopathy associated with a degue type 1 infection was diagnosed) | 2 months | Recovery |
| **Kaur R et al, 2022 ^44^** | Case report | 1M | 9 | 1 | 1 | Unconscious | Proptosis, lid edema and hemorrhagic chemosis | dorzolamide 0.3% and timolol 0.5% twice daily and Intravenous methylprednisolone 1 mg |  | Succumbed to extensive systemic involvement |
| **Dave T et al, 2022 ^45^** | Case report | 3M | 48, 21 and 34 | 3 | 2, 1, 1 | 1-Loss of vision, watering, and photophobia  2-Loss of vision, pain, watering, swelling, and redness  3-Pain, swelling, and sudden loss | 1-Intense conjunctival chemosis, microcystic corneal edema, hyphema, and raised intraocular pressure  2-echospike reflectivity in the vitreous cavity with thickened choroid and subretinal hemorrhage  3-minimal vitreous echoes with thickened choroid and a “T” sign | 1-Antiglaucoma management  2-Augmentin, intraveinous steroids  3-intraocular injection of prophylactic vancomycin and dexamethasone was performed and he was continued on the systemic antibiotics and steroids | 1-3 weeks panophtalmitis of both eyes with bacterial coinfection  2-abcess after 48h  3-panophtalmitis with bacterial coinfection | 1-Evisceration  2-Evisceration  3-Evisceration |
| **Dhoot S et al, 2022 ^46^** | Case report | 1W | 36 | 1 | 2 | Painless loss of vision | Macular striae with peripheral choroidal oedema | Topical intraocular pressure lowering drugs, cycloplegics and topical steroid and low dose of systemic steroids | Few days after | Recovery |
| **Translateur A et al, 2022 ^47^** | Case report | 1W | 70 | 1 | 2 | Bilateral vision loss | A paracentral supratemporal scotoma in the right eye and paracentral superior scotoma in the left eye. Bilateral alterations in the retino-cortical transmission. important macular function alteration at multifocal electroretinogram |  | 4 years | No recovery |
| **Malek M.I.A et al, 2022 ^48^** | Case report | 1M | 34 | 1 |  | Blurring of vision | Vitrous hemorrhage, retinal detachement | Laser hyaloidotomy, non steroidal anti-inflammatory eye drops, then pars plana vitrectomy | 6 months | Improvement |
| **Richier Q et al, 2022 ^49^** | Case report | 1W | 42 | 1 | 2 | Sudden binocular blurred visio with scotoma | Cystoid macular edema within outer plexiform and nuclear layers cystic spaces and retinal detachment | Methylprednisolone pulses for 3 days and oral prednisone for 1 month | 6 months | No improvement |

M: Men; W: Women

# Appendix 2 - Bibliography of the literature review

1. Khan N, Bhatti JM. A Case Report on Dengue Encephalitis with Optic Neuropathy. Cureus 2020; 12:e9592.

2. Haritoglou C, Dotse SD, Rudolph G, et al. A tourist with dengue fever and visual loss. Lancet Lond Engl 2002; 360:1070.

3. Li M, Zhang X, Ji Y, et al. Acute Macular Neuroretinopathy in Dengue Fever: Short-term Prospectively Followed Up Case Series. JAMA Ophthalmol 2015; 133:1329–33.

4. Nagaraj KB, Jayadev C, Yajmaan S, Prakash S. An unusual ocular emergency in severe dengue. Middle East Afr J Ophthalmol 2014; 21:347–9.

5. Pierre Filho P de TP, Carvalho Filho JP, Pierre ETL. Bilateral acute angle closure glaucoma in a patient with dengue fever: case report. Arq Bras Oftalmol 2008; 71:265–8.

6. Boo YL, Lim SY, Chin PW, Hoo FK. Bilateral optic neuritis with maculopathy: A rare manifestation of dengue fever. Malays Fam Physician Off J Acad Fam Physicians Malays 2017; 12:32–4.

7. de Amorim Garcia CA, Gomes AHB, de Oliveira ÁGF. Bilateral stellar neuroretinitis in a patient with dengue fever. Eye 2006; 20:1382–3.

8. Nainiwal S, Garg SP, Prakash G, Nainiwal N. Bilateral vitreous haemorrhage associated with dengue fever. Eye Lond Engl 2005; 19:1012–3.

9. Kanungo S, Shukla D, Kim R. Branch retinal artery occlusion secondary to dengue fever. Indian J Ophthalmol 2008; 56:73–4.

10. Puthalath AS, Samanta R, Jamil M, et al. Case Report: Foveolitis as an Indicator of Underlying Undiagnosed Dengue Fever. Am J Trop Med Hyg 2021; 104:110–4.

11. Velaitham P, Vijayasingham N. Central retinal vein occlusion concomitant with dengue fever. Int J Retina Vitr 2016; 2:1.

12. Veloso CE, Schmidt-Erfurth U, Nehemy MB. Choroidal Neovascularization Induced by Immunogenic Alteration of the Retinal Pigment Epithelium in Dengue Fever. Case Rep Ophthalmol 2015; 6:18–23.

13. Kamal R, Shah D, Sharma S, et al. Culture-positive unilateral panophthalmitis in a serology-positive case of dengue hemorrhagic fever. Indian J Ophthalmol 2018; 66:1017–9.

14. Yadav HM, Dutta Majumder P, Biswas J. Dengue associated choroiditis: a rare entity. J Ophthalmic Inflamm Infect 2017; 7:14.

15. Gupta A, Srinivasan R, Setia S, et al. Uveitis following dengue fever. Eye 2009; 23:873–6.

16. Ooi KG-J, Inglis H, Paramanathan N, et al. Dengue Fever-Associated Maculopathy and Panuveitis in Australia. Case Rep Ophthalmol Med 2016; 2016:5704695.

17. Kamoi K, Mochizuki M, Ohno-Matsui K. Dengue fever-associated necrotizing scleritis: A case report with long-term follow-up. Medicine (Baltimore) 2018; 97:e11875.

18. Juanarita J, Azmi MNR, Azhany Y, Liza-Sharmini AT. Dengue related maculopathy and foveolitis. Asian Pac J Trop Biomed 2012; 2:755–6.

19. Tabbara Khalid. Dengue retinochoroiditis. Ann Saudi Med 2012; 32:530–3.

20. Tan CSH, Teoh SCB, Chan DPL, et al. Dengue retinopathy manifesting with bilateral vasculitis and macular oedema. Eye 2007; 21:875–7.

21. Bacsal KE. Dengue-Associated Maculopathy. Arch Ophthalmol 2007; 125:501.

22. Loh B-K, Bacsal K, Chee S-P, et al. Foveolitis Associated with Dengue Fever: A Case Series. Ophthalmologica 2008; 222:317–20.

23. Akanda M, Gangaputra S, Kodati S, et al. Multimodal Imaging in Dengue-Fever-Associated Maculopathy. Ocul Immunol Inflamm 2018; 26:671–6.

24. Preechawat P, Poonyathalang A, Kurathong S, et al. Non-ischaemic Central Retinal Vein Occlusion after Dengue Viral Infection. Neuro-Ophthalmol 2012; 36:26–8.

25. Kapoor HK, Bhai S, John M, Xavier J. Ocular manifestations of dengue fever in an East Indian epidemic. Can J Ophthalmol 2006; 41:741–6.

26. Lim W, Mathur R, Koh A, et al. Ocular manifestations of dengue fever. Ophthalmology 2004; 111:2057–64.

27. Chan DPL, Teoh SCB, Tan CSH, et al. Ophthalmic Complications of Dengue. Emerg Infect Dis 2006; 12:285–9.

28. Sanjay S, Wagle AM, Au Eong KG. Optic neuropathy associated with dengue fever. Eye Lond Engl 2008; 22:722–4.

29. Tavassoli S, Carreño E, Teoh SC, et al. Optical Coherence Tomography Angiography Findings in Dengue-Related Maculopathy: A Case Report. Ophthalmic Surg Lasers Imaging Retina 2016; 47:1057–60.

30. Arya D, Das S, Shah G, Gandhi A. Panophthalmitis associated with scleral necrosis in dengue hemorrhagic fever. Indian J Ophthalmol 2019; 67:1775–7.

31. Saranappa S B S, Sowbhagya HN. Panophthalmitis in dengue fever. Indian Pediatr 2012; 49:760.

32. Shivanthan MC, Ratnayake EC, Wijesiriwardena BC, et al. Paralytic squint due to abducens nerve palsy : a rare consequence of dengue fever. BMC Infect Dis 2012; 12:156.

33. Fang PP, Pfau M, Holz FG, Finger RP. Persistent visual loss in dengue fever due to outer retinal damage. Clin Experiment Ophthalmol 2017; 45:747–9.

34. Ramananda K, Sundar M D, Mandal S, et al. Platelet Transfusion Related Panophthalmitis and Endophthalmitis in Patients with Dengue Hemorrhagic Fever. Am J Trop Med Hyg 2018; 99:1053–4.

35. Quek DTL, Barkham T, Teoh SCB. Recurrent bilateral dengue maculopathy following sequential infections with two serotypes of dengue virus. Eye Lond Engl 2009; 23:1471–2.

36. Chlebicki MP, Ang B, Barkham T, Laude A. Retinal hemorrhages in 4 patients with dengue fever. Emerg Infect Dis 2005; 11:770–2.

37. Agarwal L, Agrawal N. Retinal Vasculitis with Macular Infarction: A Dengue-related Ophthalmic Complication. Int Med Case Rep J 2020; 13:363–6.

38. Vijitha VS, Dave TV, Murthy SI, et al. Severe ocular and adnexal complications in dengue hemorrhagic fever: A report of 29 eyes. Indian J Ophthalmol 2021; 69:617–22.

39. Padmanaban DS, Jeevakala DC, Saravanan DJ, Shalini DG. Sight Threatening Ocular Complications in Dengue fever-A Prospective Study [WWW Document]. 2018.URL /paper/Sight-Threatening-Ocular-Complications-in-Dengue-Padmanaban-Jeevakala/d039ad75f8dce559373ece7b20ae53e72b0c38d8 [accessed on 20 April 2021].

40. Jyothi. Spontaneous globe rupture in dengue: A case series [WWW Document]. URL https://www.kjophthal.com/article.asp?issn=0976-6677;year=2018;volume=30;issue=2;spage=117;epage=120;aulast=Jyothi [accessed on 27 June 2021].

41. Chhavi N, Venkatesh C, Soundararajan P, Gunasekaran D. Unusual ocular manifestations of dengue fever in a young girl. Indian J Pediatr 2013; 80:522–3.

42. Chuah KH, Ng CWK, Zabri K, Wong CL. Unusual presentation of severe dengue: Dengue maculopathy. Med J Malaysia 2017; 72:73–4.

43. Guardiola GA, Villegas VM, Cruz-Villegas V, Schwartz SG. Acute macular neuroretinopathy in dengue virus serotype 1. Am J Ophthalmol Case Rep 2022; 25:101250.

44. Kaur R, Singh H, Sehgal A, Singh J. Spontaneous globe rupture: Unusual ophthalmic manifestation with dengue hemorrhagic shock syndrome. Am J Ophthalmol Case Rep 2022; 25:101297.

45. Dave TV, Sharma S, Lakshmi V, Rangaiahgari A, Murthy SI, Ali MJ, Dave VP, Pappuru RR.Evidence of dengue virus in eviscerated specimens of panophthalmitis secondary to dengue fever: A possible cause-effect phenomenon. Indian J Ophthalmol 2022; 70:965-969.

46. Dhoot SK. Bilateral Ciliochoroidal Effusion with Secondary Angle Closure and Myopic Shift in Dengue Fever. Ocul Immunol Inflamm 2022; 24:1-4.

47. Translateur A, Perez-Rueda M. Acute macular neuroretinopathy associated to dengue disease. Am J Ophthalmol Case Rep 2022; 26:101474.

48. Malek MA, Niyonzima JC, Pathan MAHK, Rahman MM. Pars Plana Vitrectomy for a Sub-Internal Limiting Membrane Hemorrhage and Vitreous Hemorrhage Secondary to Dengue Fever: A Case Report. Cureus 2022; 14:e25916.

49. Richier Q, Bataille N, Gauzëre L, Safla I, Villeroy F, Raffray L. Dengue-related maculopathy. J Travel Med 2022; taac106.
